# Author Correction: Atomically precise graphene etch stops for three dimensional integrated systems from two dimensional material heterostructures

**DOI:** 10.1038/s41467-018-07297-5

**Published:** 2018-11-20

**Authors:** Jangyup Son, Junyoung Kwon, SunPhil Kim, Yinchuan Lv, Jaehyung Yu, Jong-Young Lee, Huije Ryu, Kenji Watanabe, Takashi Taniguchi, Rita Garrido-Menacho, Nadya Mason, Elif Ertekin, Pinshane Y. Huang, Gwan-Hyoung Lee, Arend M. van der Zande

**Affiliations:** 1 Department of Mechanical Science and Engineering, University of Illinois at Urbana-Champaign, 1206 W Green Street, Urbana, IL 61801 USA; 2 Department of Materials Science and Engineering, Yonsei University, 50 Yonsei-ro, Seodaemun-gu, Seoul 03722 Korea; 3 Department of Physics, University of Illinois at Urbana-Champaign, 1110 W Green Street, Urbana, IL 61801 USA; 4 National Institute for Materials Science, 1-1 Namiki, Tsukuba, Ibaraki 305-0044 Japan; 5 Frederick Seitz Materials Research Laboratory, University of Illinois at Urbana-Champaign, 104 S Goodwin Avenue MC-230, Urbana, IL 61801 USA; 6 Department of Materials Science and Engineering, University of Illinois at Urbana-Champaign, 1304 W Green Street, Urbana, IL 61801 USA

Correction to: *Nature Communications*; 10.1038/s41467-018-06524-3; published online 28 September 2018

The original version of this Article contained an error in the second sentence of the second paragraph of the ‘Electrical properties of fluorinated graphene contacts’ section of the Results, which incorrectly read ‘The mobility was calculated by the Drude model, *μ* = *ne*/*σ* where *μ*, *n*, *e*, and *σ* are the carrier mobility, carrier density, electron charge, and sheet conductivity, respectively’. The correct version states ‘*μ* = *σ/ne* ’ in place of ‘*μ* = *ne*/*σ* ’. This has been corrected in both the PDF and HTML versions of the Article.

