# Supplementary material for: Atomically precise graphene etch stops for three dimensional integrated systems from two dimensional material heterostructures
Source: Nat Commun. 2018 Sep 28;9:3988. doi: 10.1038/s41467-018-06524-3 (PMC6162276; doi:10.1038/s41467-018-06524-3)
Supplement: Supplementary file 1 — Supplementary Information [file 41467_2018_6524_MOESM1_ESM.pdf]

Supplementary Information

Atomically precise graphene etch stops for three dimensional integrated systems from two dimensional material heterostructures

Son et al.

Supplementary Figures

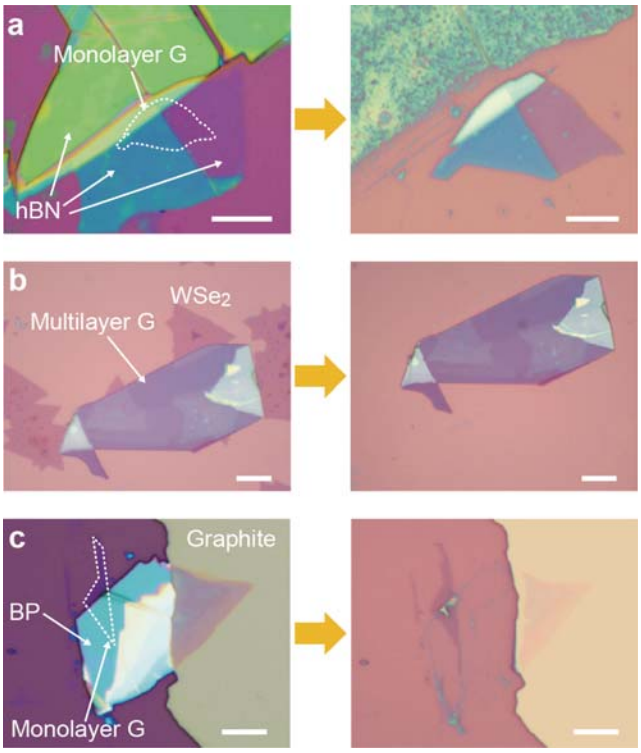

Supplementary Figure 1 XeF_2 etching behaviors of various 2D materials protected by graphene. Optical microscope images of **a** hBN, **b** WSe_2, and **c** BP covered by graphene before and after XeF_2 etching (all scale bars are 10 μm). All of these images were taken under the same etch time of 30 seconds at 3 Torr. Noted that lateral etching occurs in BP covered by graphene due to the high etching rate of BP.

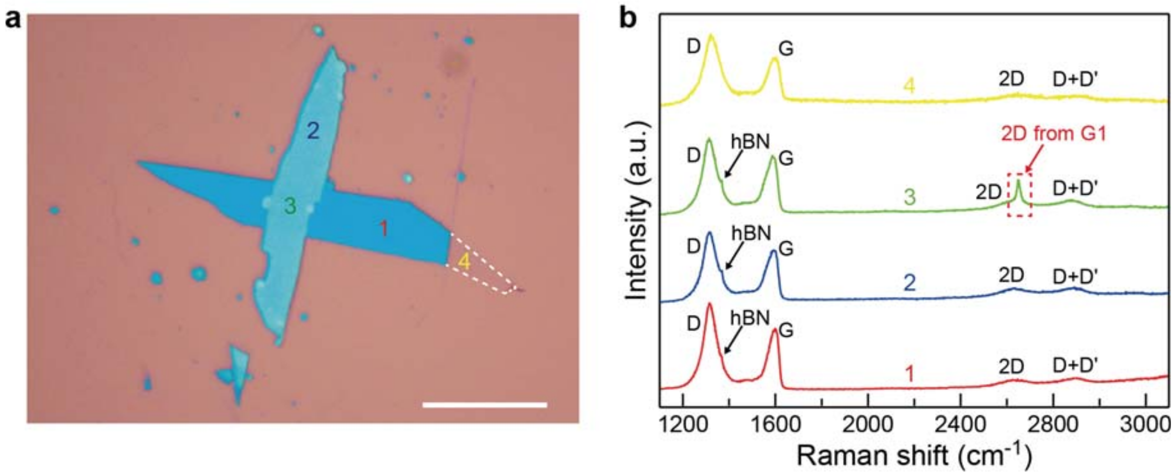

Supplementary Figure 2 Etching of hBN/G/hBN/G/hBN heterostructure by XeF_2. **a** Optical image (the scale bar is 10 μm) and **b** Raman spectra of heterostructure after XeF_2 etching. After exposure to XeF_2, Raman spectra were obtained from point 1 (FG1/hBN1), point 2 (FG2/hBN2/hBN1), point 3 (FG2/hBN2/G1/hBN1), and point 4 (FG1) of the sample in Figure 1c. The hBN region sitting on graphene was etched away and hBN region under graphene remained unchanged. Raman spectra of point 1, 2, and 4 show that the graphene exposed to XeF_2 has increased D peak and decreased 2D peak, indicative of fluorination. However, the point 3, which is protected by top graphene (G2), clearly showed distinct Raman signals of pristine graphene located in the middle (G1) as indicated by a square of red dashed line. This means that the impermeable top-FG can prevent further etching of hBN and fluorination of embedded graphene under FG.

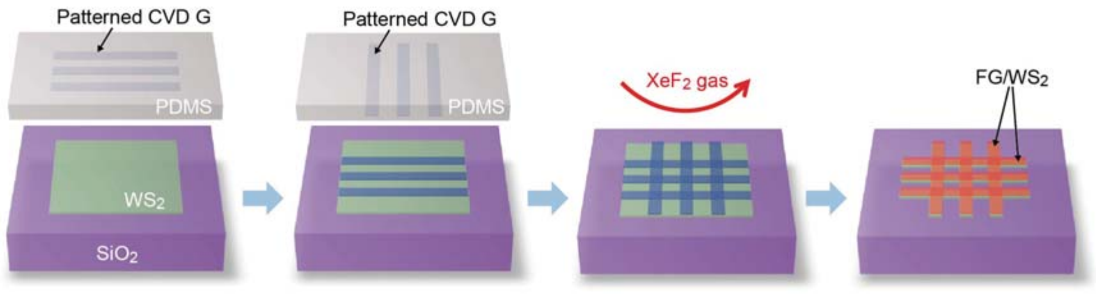

Supplementary Figure 3 Large scale patterning of monolayer WS_2 film using pre-patterned graphene etch stops (GES). Pre-patterned CVD-grown GES are sequentially transferred onto CVD-grown monolayer WS_2 with perpendicular direction relative to each other, resulting in cross-hatch pattern. After XeF_2 etching, uncovered WS_2 regions are etched away, while WS_2 regions protected by GES remain unchanged. The graphene exposed to XeF_2 turns to insulating FG.

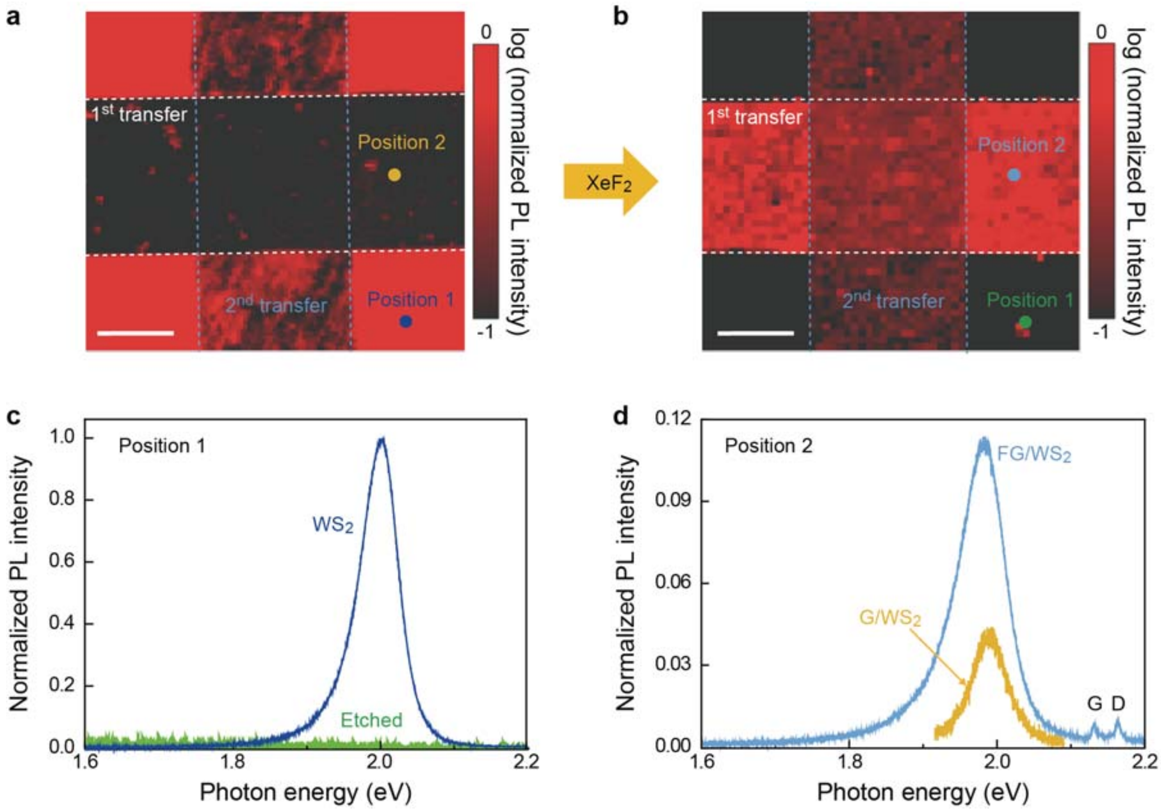

Supplementary Figure 4 Effect of FG on optical properties of WS_2. PL mapping images of the patterned WS_2 sheet (Figure 2a in main text) **a** before and **b** after XeF_2 gas exposure (the scale bars are 10 μm). Positions of 1 and 2 in **a** indicate the regions of WS_2 and G/WS_2 before XeF_2 exposure. After XeF_2 exposure, WS_2 in position 1 is etched and G/WS_2 in position 2 changes to FG/WS_2. Non-uniform PL intensity in two regions (1st transfer and 2nd transfer) is probably due to polymer residues and small cracks of graphene induced during wet transfer of pre-patterned graphene. Nevertheless, it should be noted that PL of WS_2 is quenched under graphene, meanwhile PL of WS_2 is restored after XeF_2 exposure. **c** PL spectra of WS_2 in position 1 before and after XeF_2 etching. Absence of PL intensity after XeF_2 etching shows that WS_2 uncovered by

graphene is completely etched away. **d** PL spectra of graphene-covered WS_2 in position 2 before and after XeF_2 etching. The G/WS_2 region shows quenching of PL intensity due to transfer of generated excitons from WS_2 to conducting graphene. Meanwhile, PL intensity of WS_2 protected by FG is enhanced by more than twice after XeF_2 etching, due to no more charge transfer from WS_2 to insulating FG.

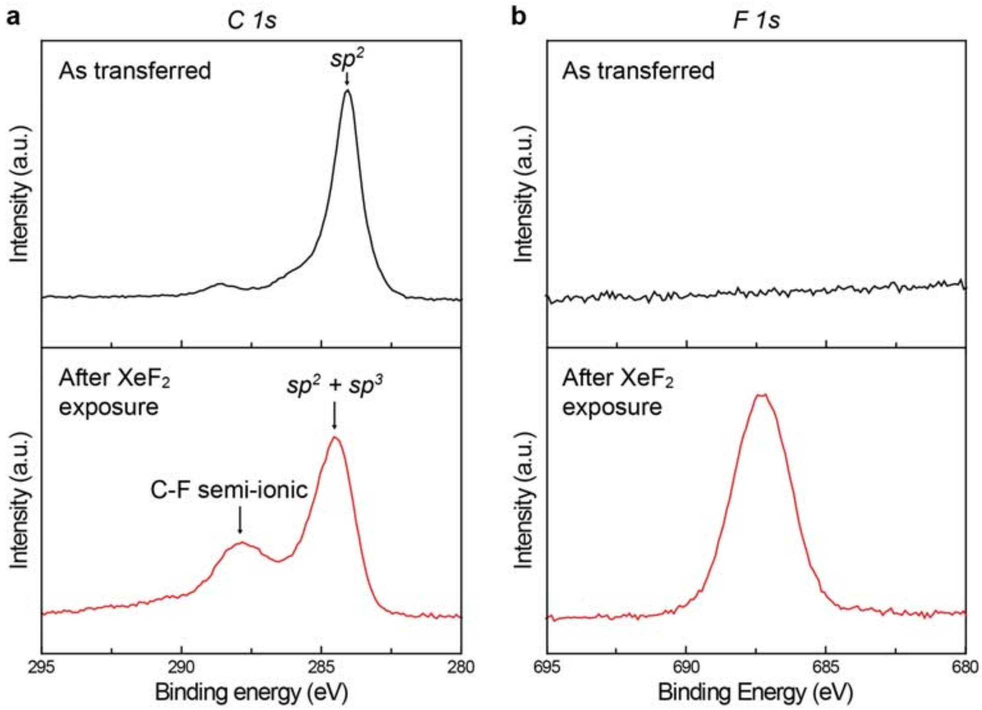

Supplementary Figure 5 XPS analysis on fluorinated CVD graphene. XPS spectra of **a** the *C 1s* peak and **b** F 1s peak in CVD graphene on Au-coated SiO_2 substrate before (black curves) and after XeF_2 etching (red curves). The sp^2 bond peak in *C 1s* centered at 284.6 eV is blue-shifted and broadened after XeF_2 etching. The emergence of C-F semi-ionic peak at 287.6 eV shows that sp^3 bonds of C-F were generated on the surface of graphene[1]. The core level peak of *F 1s* was observed in **b** after XeF_2 etching, indicating that substantial amount of F is chemically bonded to the surface of graphene. The calculated C–F atomic ratio was 1:0.22, which means that graphene was highly fluorinated to 22%, close to the fluorination limit of single sided FG of 25%. Absence of other peaks shows that there are no other defects or bonds.

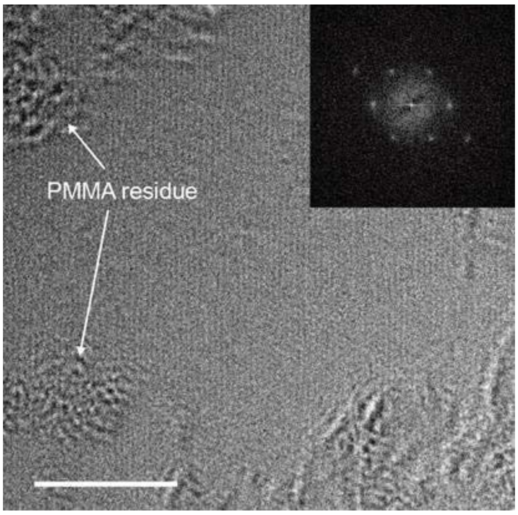

Supplementary Figure 6 HR-TEM image of FG. The HR-TEM image shows crystal lattice of CVD graphene fluorinated by exposure to XeF_2 for 100 seconds (the scale bar is 5 nm). The CVD graphene was transferred onto a TEM grid with PMMA transfer method, followed by annealing (340°C, 4 hours) to remove PMMA residue. However, small amounts of PMMA residue are still observed as indicated by arrows. After exposure to XeF_2 gas, no voids were observed in the fluorinated region. The Fast Fourier Transform (FFT) in the inset shows that fluorinated graphene maintains crystallinity after fluorination. This confirms that fluorination process makes no voids in FG and FG can be used as an impermeable membrane to the XeF_2.

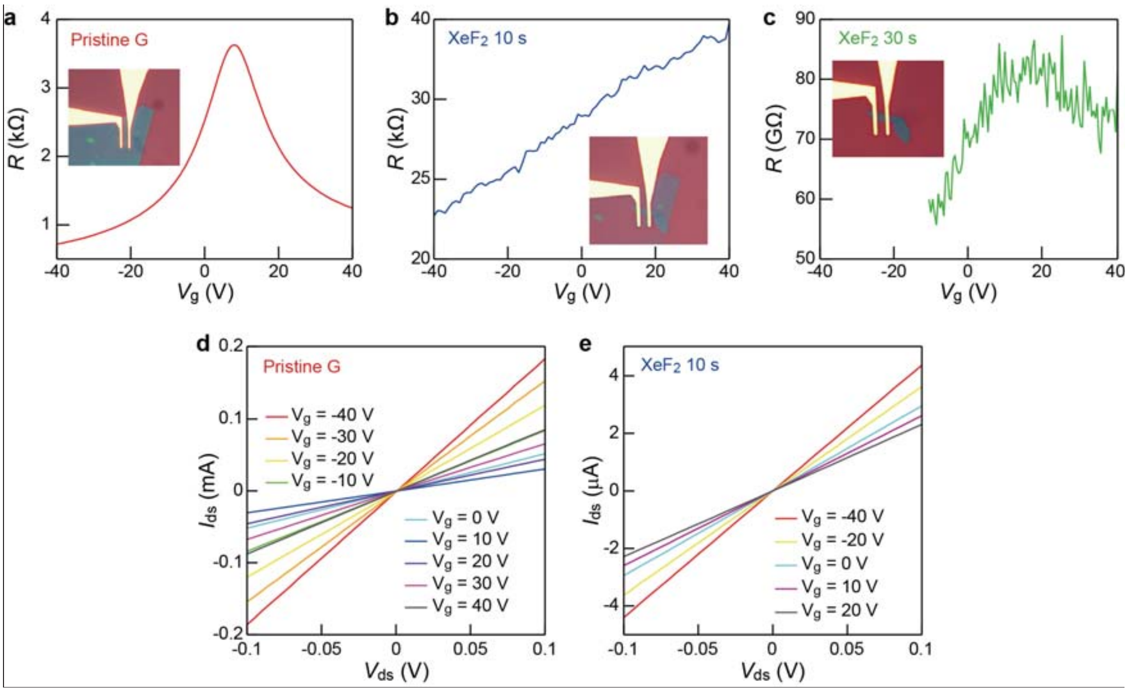

Supplementary Figure 7 Electrical measurement of FG channel. Resistance *vs.* gate voltage curves of **a** pristine graphene, **b** FG treated with XeF_2 for 10 seconds, and **c** FG treated with XeF_2 for 30 seconds. Fluorination makes graphene p-doped and fully FG is insulating. Output (I_{ds}–V_{ds}) curves of **d** pristine graphene and **e** FG treated with XeF_2 for 10 seconds.

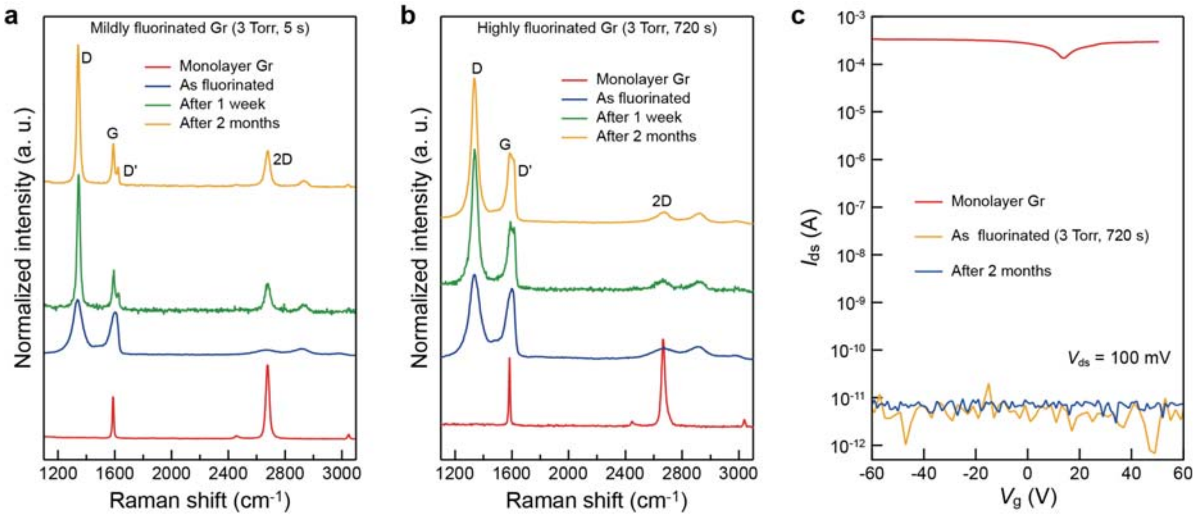

Supplementary Figure 8 The stability of FG. To measure the stability over time of fluorination, graphene on hBN is exposed to XeF_2 under two conditions: mild (3 torr, 5 seconds) and high (3 torr, 720 seconds). **a, b** The Raman signals of the mild and high conditions respectively before exposure, immediately after exposure, after one week, and after 2 months. While the Raman signature of the mild FG degrades after several days with a return of the 2D peak. The high FG Raman signature remains stable for at least 2 months in ambient conditions. **c** Electrical transport measurements of an FET made from the high FG before exposure, directly after exposure and after 2 months. The high FG maintains electrical insulating behavior for at least 2 months in ambient conditions.

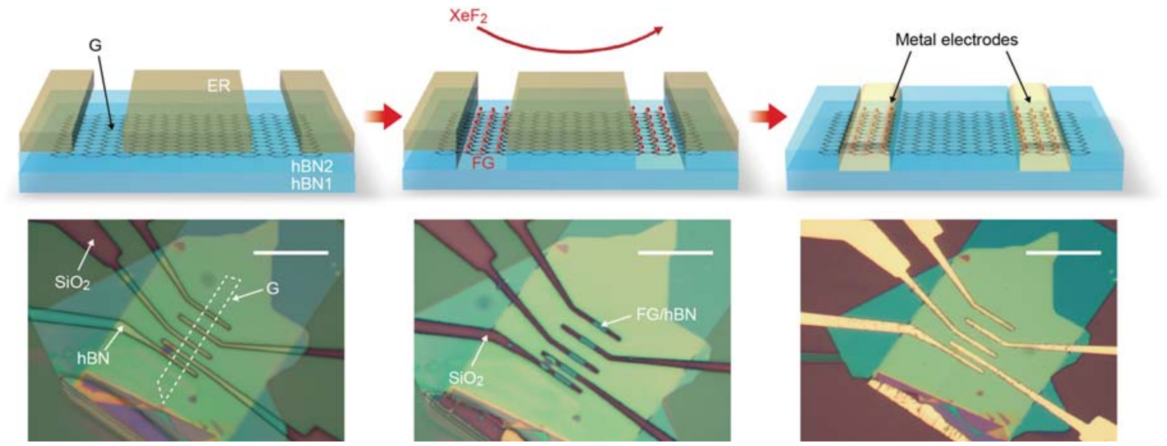

Supplementary Figure 9 One-step fabrication process for hBN-encapsulated graphene devices with FG via contacts. Electrode patterns of PMMA on a stack of hBN/G/hBN were generated by e-beam lithography. After XeF_2 etching of 30 seconds in 3 Torr, hBN region on graphene in the exposed regions was etched away and graphene was fluorinated. The direct contact to the exposed FG regions was produced by deposition of metals (Cr/Pd/Au) through the same lithographic mask after etching. The metallized FG is never directly exposed to solvents or polymers. Following metallization, the mask is lifted off in acetone. All scale bars are 10 μm.

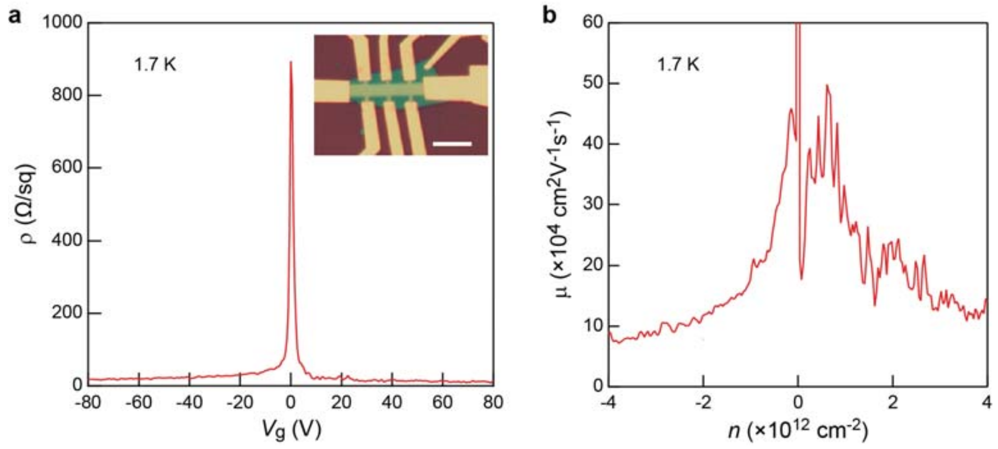

Supplementary Figure 10 Low temperature measurement of electrical properties in hBN-encapsulated graphene device with FG via contacts. **a** Transfer curve of 4-point resistivity versus gate voltage of the hBN-encapsulated graphene Hall bar device with FG via contacts, measured at 1.7 K with I_{ds} = 100 nA. The inset shows optical image of the device (the scale bar is 5 μm). **b** Carrier mobility as a function of carrier density extracted from the resistivity measurement by applying the Drude model.

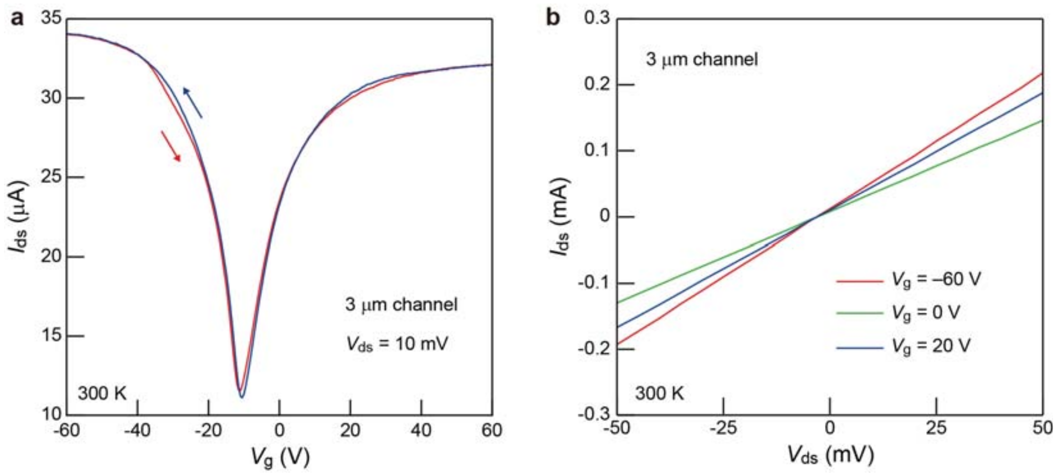

Supplementary Figure 11 Electrical measurement of the graphene TLM device. **a** Transfer curves (I_{ds}–V_g) and **b** output curves (I_{ds}–V_{ds}) of 3 μm-channel graphene device in the TLM device of Figure 3 (in the main text) at room temperature. There is no hysteresis in the transfer curve due to the absence of trapped charges at the interfaces and in the hBN encapsulation. The linearity in output curves indicates that the contact resistance of the device is Ohmic.

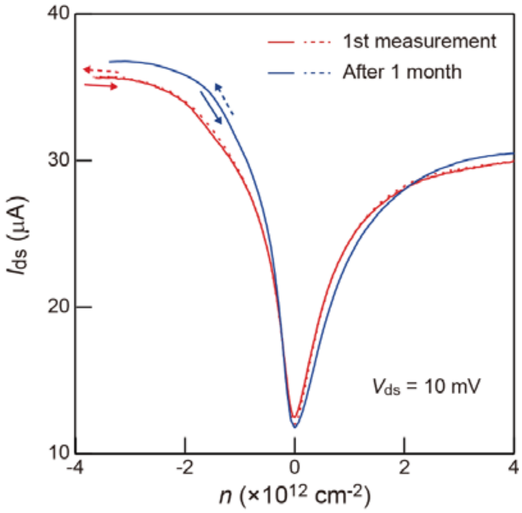

Supplementary Figure 12 Stability of the embedded graphene FET with FG via contacts. Transfer curves (I_{ds}–V_g) of the hBN-encapsulated graphene device with FG via contacts both directly after fabrication (red) and after 1 month (blue). The device was kept in ambient condition for a month after first measurement. While it is already established that graphene encapsulated by hBN is stable for long periods, this measurement indicates that the FG contacts are also stable.

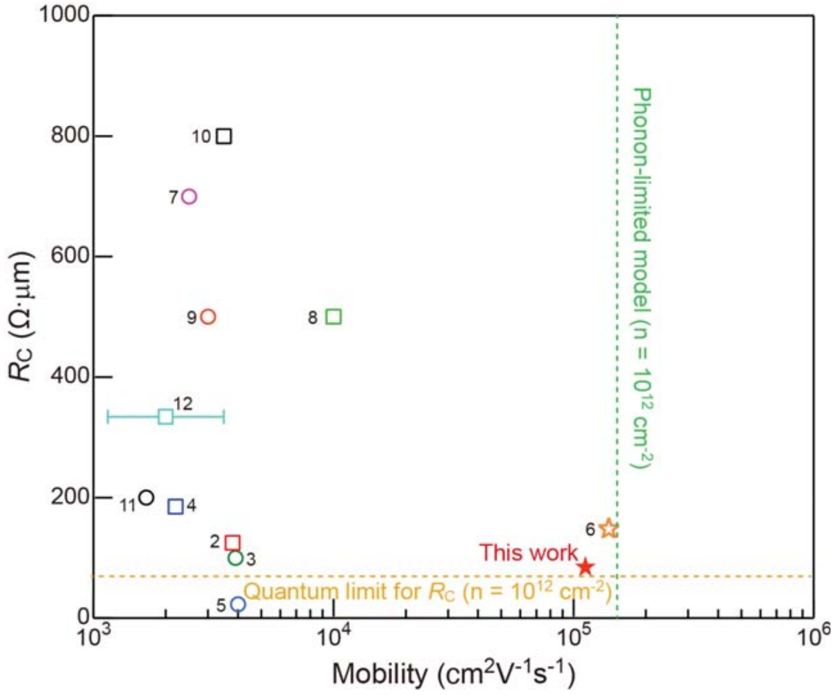

Supplementary Figure 13 Comparison of device performances. Figure shows contact resistance (R_C) *vs.* mobility of various researches, and its specific information are described in Supplementary Table 2. The hBN-encapsulated graphene device with FG via contacts shows superior properties in terms of carrier mobility and contact resistance, compared to various graphene devices reported previously. Our devices show the highest mobility and lowest contact resistance, close to their quantum limits[6, 13, 14].

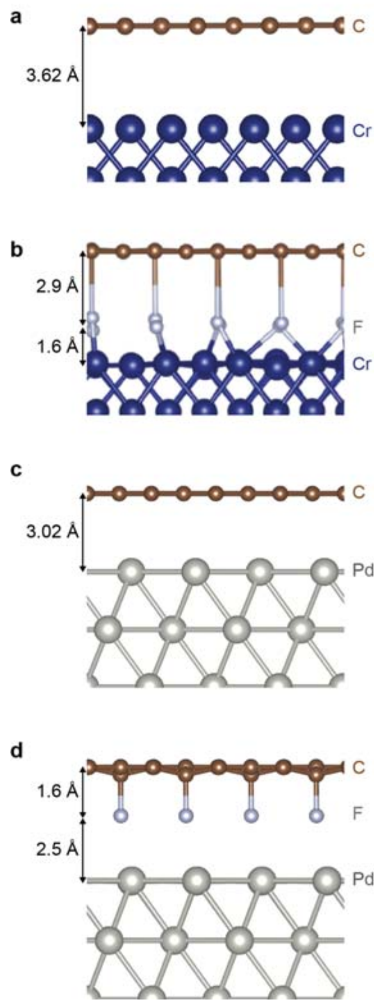

Supplementary Figure 14 Simulation of interfacial properties in FG-metal. Atomic structures at the interfaces between **a** G-Cr, **b** FG-Cr, **c** G-Pd, and **d** FG-Pd. It was assumed that (111) surface of metal is parallel to the basal plane of graphene or FG (fluorinated by 25%). When metals were placed on pristine graphene, atomic distances of C-Cr and C-Pd are as large as 3.62 Å and 3.02 Å, respectively. However, both Cr and Pd on FG showed smaller atomic distances of C-F-Cr and C-F-Pd.

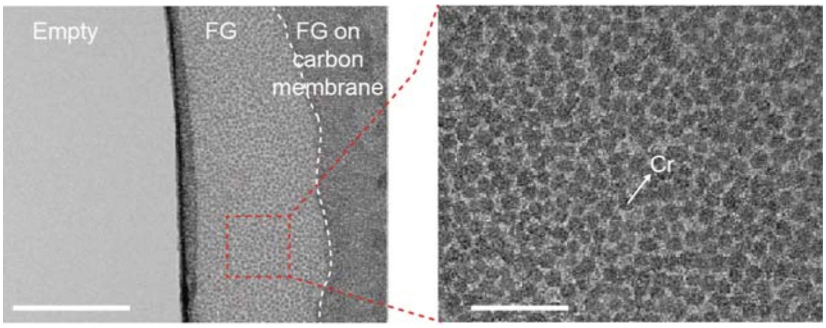

Supplementary Figure 15 TEM observation of Cr islands deposited on FG. TEM images of FG deposited by Cr (the scale bars are 100 nm and 20 nm, respectively). To confirm the shape of Cr coating, Cr of 1 nm was deposited on fluorinated CVD graphene. The deposited Cr, used as adhesion layer, forms islands on FG rather than uniform film, which means that, in the FG via contact region of our device, both Cr and Pd are in contact with FG because Cr (1 nm) and Pd (30 nm) are subsequently deposited on FG.

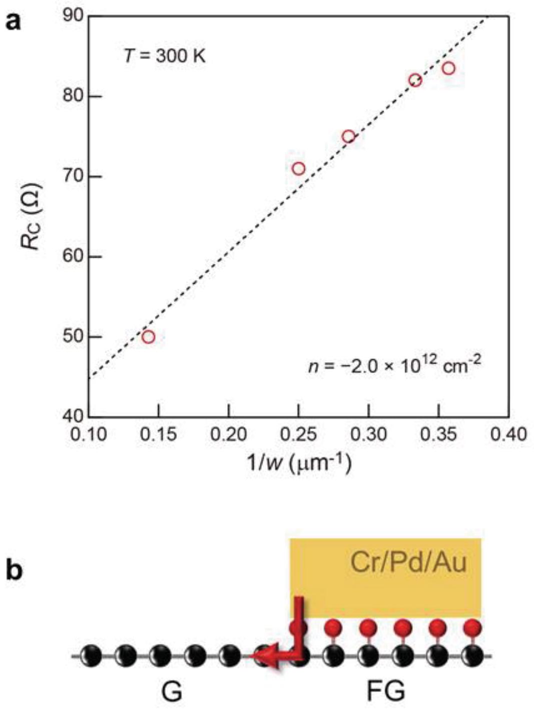

Supplementary Figure 16 Relation between contact resistance and contact width. **a** Contact resistance *vs.* channel widths measured from five devices. **b** Schematic illustration of charge transfer in FG via contact. The contact resistance is linearly proportional to the channel width, which means that the dominant resistance for charge injection is at the one-dimensional interface between graphene and FG, rather than the two dimensional surface contact between the metal and the FG.

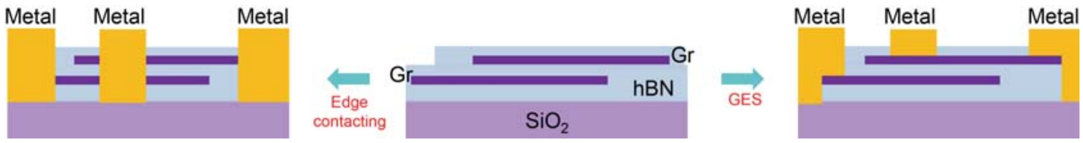

Supplementary Figure 17 Comparison of patterning using edge contacts and GES. Edge contacts require careful offsetting of each active layer because the etching is not selective so all vertically aligned layers in the heterostructure are exposed simultaneously. In contrast, the GES technique allows selective and separate contact to vertically offset layers because it will stop on the topmost graphene layer.

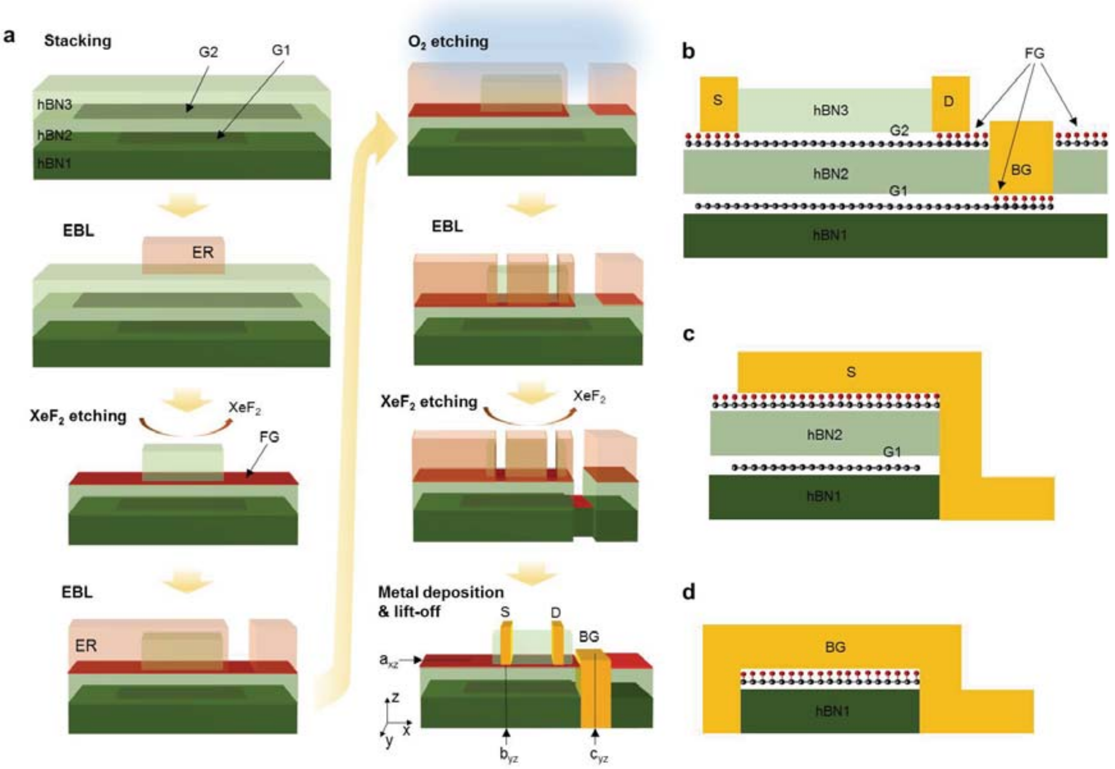

Supplementary Figure 18 Fabrication process of hBN-encapsulated graphene device with graphene backgate. **a** Schematic illustrations describing fabrication of the device in detail. Starting from hBN/G/hBN/G/hBN heterostructure, e-beam lithography (EBL) was carried out to define channel, followed by XeF_2 etching. To form via contact to the bottom graphene, the top graphene mask must be broken, so a smaller opening on the top FG was patterned with EBL then etched with O_2 plasma at 50 W for 10 seconds. To access the bottom graphene layer and to pattern the electrodes on both the top and bottom layers, a final EBL patterning step followed by a final XeF_2 etch step is used, then metal is deposited in the patterned regions, which now separately access both the bottom and top graphene layers. The metal is then lifted off in solvents

to form source and drain contacts to the top graphene and gate contact to the bottom graphene. **b-d** Schematics of cross-section of the resultant device. a_{xz}, b_{yz}, and c_{yz} indicate plane views of xz along x, yz at source electrode, and yz at BG electrode, respectively.

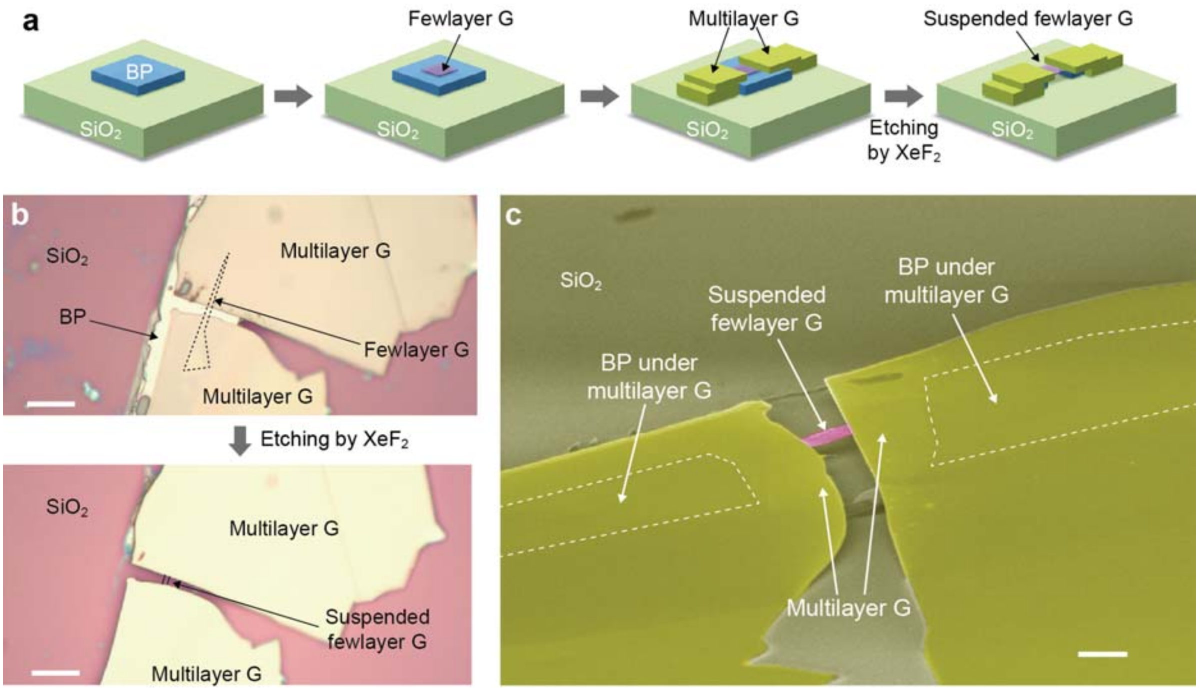

Supplementary Figure 19 Fabrication process of a graphene resonator. **a** Schematics of fabrication process for graphene resonator of Figure 4e in the main text. **b** Optical microscopic images of the stack before and after XeF_2 etching (the scale bars are 10 μm). **c** False-color SEM image of the fabricated graphene resonator (the scale bar is 2 μm). For fabrication of graphene resonator, a thick BP, which has high etching rate, was first exfoliated on SiO_2 substrate. A narrow few-layer graphene and two thick and large graphene flakes were subsequently transferred on the BP flake. Thick graphene flakes are separated by 2 μm, which corresponds to the length of the suspended few-layer graphene. After one-step exposure of the stack to XeF_2 gas, the BP region under narrow few-layer graphene was rapidly etched away, whereas the BP under two large graphene flakes remained. The result is a suspended graphene membrane over a SiO_2 substrate obtained using a dry etch process.

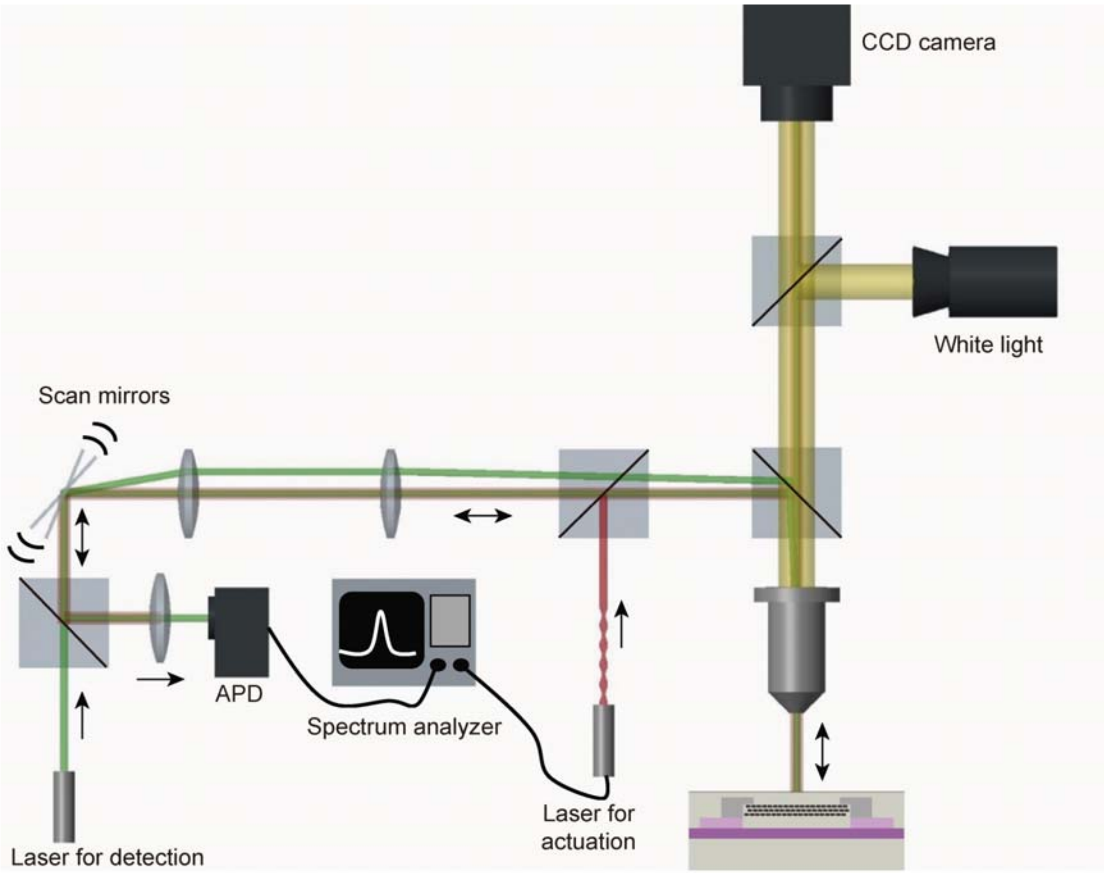

Supplementary Figure 20 Schematic illustration of optical interferometry apparatus for measurement of graphene resonator. The optical interferometry was used to actuate and detect the motion of the graphene membrane. Two lasers (637 nm and 520 nm) were focused on the center of the resonator. The 637 nm laser was modulated by applying MHz frequency oscillating voltage with a spectrum analyzer. This modulation will heat up and cool down the membrane, leading to the membrane actuation at the drive frequency due to the thermal expansion. The mechanical motion is then detected by measuring the dynamic optical reflectance of a 520 nm diode laser. Due to Fabry-Pérot interferometry, the reflected light is modulated by the change in position of the membrane with respect to the reflecting silicon backgate. The change in reflected

light was monitored by photodetector (APD) and Fourier-transformed by spectrum analyzer. The measurement was performed in an optical cryostat at room temperature, in vacuum with pressure less than 5×10^{-6} Torr.

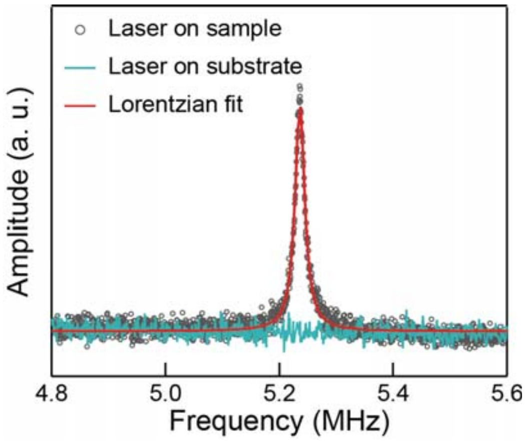

Supplementary Figure 21 Graphene Resonance. The measured dynamic reflected laser response depends on whether the laser is focused on the graphene membrane or on the substrate. Because the resonance only appears when on the suspended region, it must be a mechanical resonance and not a spurious electrical resonance.

Supplementary Tables

	Black phosphorus (BP)	MoS_2	WSe_2	Hexagonal boron nitride (hBN)
Etching rate (nm/sec)	18.5	4.1	4.0	3.4

Supplementary Table 1 Etching rates of 2D materials. Etching rate of BP is much higher than those of other 2D materials of MoS_2, WSe_2, and hBN.

	Ref.	Metal	Dielectric /substrate	Treatment	Contact	Encapsulation
□	2	Cu, Pd	HfO_2 / SiC	–	Patterning	–
○	3	Ni	SiO_2	–	Ni-etched	–
□	4	Pd/Au	SiO_2	–	–	–
○	5	Cu, Ti, Pd	PVP/PMF	n-doping	Edge patterning	–
☆	6	Cr/Pd/Au	hBN/SiO_2	–	Edge contact	○
○	7	Ti, Ag, Co, Cr, Fe, Ni, Pd	SiO_2	–	–	–
□	8	Cr/Au, Ti/Au, Ni	SiO_2	–	–	–
○	9	Ni, Au, Pd, Ni/Au, Pt/Au	SiO_2	–	–	–
□	10	Ni on fewlayer graphene	SiO_2	–	–	–
○	11	Ti/Au	SiO_2	Ultraviolet ozone	–	–
□	12	Ni	SiO_2	PMMA free & annealing	–	–
★	This work	Cr/Pd/Au	hBN/SiO_2	XeF_2 gas	via contact	○

Supplementary Table 2 Specific information of the devices described in Supplementary Figure 13.

Supplementary Notes

Supplementary Note 1 XeF_2 etching mechanism of 2D materials. XeF_2 gas etched all the 2D materials tested in our work other than graphene, as shown in Supplementary Figure 1. However, etching rates of 2D materials are different: BP > MoS_2 ≈ WSe_2 > hBN (see Supplementary Table 1). As we demonstrated in Figure 4e, different etching rate of each 2D layers can be utilized to fabricate complicated multilayer and suspended geometries. For various 2D materials, such as MoS_2, WSe_2, hBN, and BP, the chemical equations occurring during XeF_2 etching are[15];

$$MoS_2 + 8XeF_2 \rightarrow 8Xe + MoF_4 + 2SF_6$$

$$WSe_2 + 8XeF_2 \rightarrow 8Xe + WF_4 + 2SeF_6$$

$$2BN + 3XeF_2 \rightarrow 2BF_3 + 3Xe + N_2$$

$$2P + XeF_2 \rightarrow 2PF + Xe$$

Many of these byproducts are toxic. For example, when hBN is etched with XeF_2 gas, boron trifluoride (BF_3), which is a pungent colorless toxic gas, is generated. Therefore, we carried out all etching processes in the clean room equipped with chemical exhaust capabilities.

Supplementary References

1. Samanta, K., Some, S., Kim, Y., Yoon, Y., Min, M., Lee, S. M., Park, Y. & Lee, H. Highly hydrophilic and insulating fluorinated reduced graphene oxide. *Chem. Commun.* **49**, 8991–8993 (2013).
2. Smith, J. T., Franklin, A. D., Farmer, D. B. & Dimitrakopoulos, C. D. Reducing contact resistance in graphene devices through contact area patterning. *ACS Nano* **7**, 3661–3667 (2013).
3. Leong, W. S., Gong, H. & Thong, J. T. L. Low-contact-resistance graphene devices with nickel-etched-graphene contacts. *ACS Nano* **8**, 994–1001 (2014).
4. Xia, F., Perebeinos, V., Lin, Y., Wu, Y. & Avouris, P. The origins and limits of metal–graphene junction resistance. *Nat. Nanotechnol*. **6**, 179–184 (2011).
5. Park, H. Y., Jung, W. S., Kang, D. H., Jeon, J., Yoo, G., Park, Y., Lee, J., Jang, Y. H., Lee, J., Park, S., Yu, H. Y., Shin, B., Lee, S. & Park, J. H. Extremely low contact resistance on graphene through n-type doping and edge contact design. *Adv. Mater*. **28**, 864–870 (2016).
6. Wang, L., Meric, I., Huang, P. Y., Gao, Q., Gao, Y., Tran, H., Taniguchi, T., Watanabe, K., Campos, L. M., Muller, D. A., Guo, J., Kim, P., Hone, J., Shepard, K. L., Dean, C. R. One-dimensional electrical contact to a two-dimensional material. *Science* **342**, 614–617 (2013).
7. Watanabe, E., Conwill, A., Tsuya & Koide, D. Y. Low contact resistance metals for graphene based devices. *Diam. Relat. Mater*. **24**, 171–174 (2012).

8. Nagashio, K., Nishimura, T., Kita, K. & Toriumi, A. *Electron Devices Meeting (IEDM), IEEE International* (2009).
9. Gahoi, A., Wagner, S., Bablich. A., Kataria, S., Passi, V., Lemme, M. C. Contact resistance study of various metal electrodes with CVD graphene. *Solid State Electron.* **125**, 234–239 (2016).
10. Venugopal, A. Colombo, L. & Vogel, E. M. Contact resistance in few and multilayer graphene devices. *Appl. Phys. Lett.* **96**, 013512 (2010).
11. Li, W., Liang, Y., Yu, D., Peng, L., Pernstich, K. P., Shen, T., Walker, A. R., Cheng, G., Hacker, C. A., Richter, C. A., Li, Q., Gundlach, D. J., Liang, X. Ultraviolet/ozone treatment to reduce metal-graphene contact resistance. *Appl. Phys. Lett.* **102**, 183110 (2013).
12. Leong, W. S., Nai, C. T. & Thong, J. T. L. What Does Annealing Do to Metal–Graphene Contacts? *Nano Lett.* **14**, 3840–3847 (2014).
13. Allain, A. Kang, J. Banerjee, K. & Kis, A. Electrical contacts to two-dimensional semiconductors. *Nat. Mater.* **14**, 1195–1205 (2015).
14. Landauer, R. Spatial variation of currents and fields due to localized scatterers in metallic conduction. *IBM J. Res. Dev*. **1**, 223–231 (1957).
15. Huang, Y., Wu, J., Xu, X., Ho, Y., Ni, G., Zou, Q., Koon, G. K. W., Zhao, W., Castro Neto, A. H., Eda, G., Shen, C., Özyilmaz, B. An innovative way of etching MoS_2: Characterization and mechanistic investigation. *Nano Res*. **6**, 200–207 (2013).

- End of Supplementary Information -
